# Association of Polyamine Intake, Other Dietary Components, and Fecal Content of *N*-acetyl Putrescine and Cadaverine with Patients’ Colorectal Lesions

**DOI:** 10.3390/nu16172894

**Published:** 2024-08-29

**Authors:** Eva Barreiro-Alonso, Paula Castro-Estrada, Manuel Sánchez, Pablo Peña-Iglesias, Lorena Suárez, Begoña Cantabrana

**Affiliations:** 1 Instituto Universitario de Oncología del Principado de Asturias (IUOPA), 33006 Oviedo, Spain; evabarreiroalonso@yahoo.es (E.B.-A.); bego@uniovi.es (B.C.); 2 Instituto de Investigación Sanitaria del Principado de Asturias (ISPA), 33011 Oviedo, Spain; 3 Servicio de Digestivo, Hospital Universitario Central de Asturias (HUCA), 33011 Oviedo, Spain; 4 Farmacología, Departamento de Medicina, Universidad de Oviedo, 33006 Oviedo, Spain

**Keywords:** colorectal cancer, polyamine dietary intake, feces *N*-acetyl putrescine, feces cadaverine

## Abstract

Colorectal cancer (CRC) is the second leading cause of cancer death worldwide. Early detection and the modification of risk factors, such as diet, can reduce its incidence. Among food components, polyamines are important for maintaining gastrointestinal health and are metabolites of gut microbiota. Their disruption is linked to CRC, making polyamines a potential marker of the disease. This study analyzed the relationship between dietary components, including polyamines, and the presence of polyamines in feces to determine whether their presence could contribute to predicting the occurrence of colorectal lesions in patients. In total, 59 participants of both sexes (aged 50 to 70 years) who had undergone colonoscopy screening for CRC (18 without and 41 with colorectal lesions) participated in the study. A nutritional survey and determination of fecal polyamine content were performed. Specific dietary components and putrescine levels were higher in patients with colorectal lesions. The diet ratio of putrescine–spermidine and the fecal content of *N*-acetyl putrescine and cadaverine were elevated in patients with precancerous lesions and adenocarcinomas, showing a potential predictive value for the presence of colorectal lesions. These findings suggest that *N*-acetyl putrescine and cadaverine could be complementary markers for the diagnosis of suspected colorectal lesions.

## 1. Introduction

Colorectal cancer (CRC) is the third most prevalent cancer and the second leading cause of cancer-related death worldwide [1]. It can be prevented by modifying risk factors, such as diet and lifestyle, especially in cases of genetic mutations and immune disorders [2]. Early detection based on effective screening programs and the removal of precancerous lesions are crucial in preventing the disease, and they have decreased its incidence in high-income countries [1].

Certain dietary nutrients should be avoided or limited, as they may be directly harmful or negatively affect the microbiota and its metabolites, which have pro-inflammatory properties and may lead to tumorigenesis if maintained over time [3]. By contrast, consumption of other foods should be encouraged, such as whole grains, non-starchy vegetables, and raw fruits, which are rich in fiber and phytonutrients and have anti-inflammatory and antioxidant properties. The Dietary Inflammatory Index (DII) can be used to assess the inflammatory capacity of diets [4].

Among essential components of foods, polyamines (putrescine, spermidine, and spermine) are important for maintaining essential physiological functions, including that of the epithelium of the gastrointestinal mucosa, which has a high need for polyamines due to its rapid turnover. Polyamines are produced by endogenous synthesis and intestinal uptake, including from diet (as they are contained to a greater or lesser degree in all foods) [5], from biliary and pancreatic exocrine secretion, from cell exfoliation, and from intestinal microbiota.

Evidence suggests that ingested polyamines play a role in the growth and healing of the gastrointestinal mucosa [6], stimulate the immune system, and modulate spontaneous gastrointestinal motility [7], facilitating the digestion and absorption of nutrients. The characteristics of diets vary between different countries as well as between individuals in the same community, leading to variations in polyamine intake and their association with inflammatory dietary properties [5]. Furthermore, polyamines are linked to colorectal carcinogenesis, giving rise to the therapeutic strategy of controlling exogenous polyamines in addition to chemoprevention with drugs to inhibit endogenous polyamine synthesis, such as difluoromethylornithine (eflornithine) [8].

Nutrients and other essential components of foods, including polyamines, contribute to maintaining gut microbiota homeostasis [3,9,10]. Diverse causes may produce microbiota dysbiosis, which is closely associated with intestinal epithelial cells’ sensitization and with carcinogenesis development [10]. Some bacteria have been identified as prevalent in CRC patients. Among them, the enterotoxigenic Bacteroides fragilis, Escherichia coli, and Fusobacterium nucleatum are relevant, and the mechanisms involved in the pathogenesis of inflammation and carcinogenesis have been reviewed [11,12,13,14,15]. Among other influences, the physiological and pathological actions of the microbiota are produced by multiple metabolites, including polyamines. The implementation of noninvasive screening, such as via fecal occult blood tests, together with colonoscopy, enables the early diagnosis of precancerous lesions, hence preventing and reducing cases of CRC.

Our research investigated the relationship between dietary intake components, including polyamines, and the presence of biogenic amines in the feces of patients with colorectal lesions identified by colonoscopy. The lesions were categorized according to their pathological anatomy, with hyperplastic lesions considered benign and precancerous lesions (tubular and tubulovillous adenomas) and adenocarcinomas deemed malignant. Additionally, we aimed to determine whether the presence of biogenic amines could predict the occurrence of colorectal lesions in patients.

## 2. Material and Methods

### 2.1. Participants, Study Design, and Variables

A descriptive cross-sectional study was conducted on 59 Caucasian volunteers between the ages of 40 and 70 who were recruited consecutively from September 2019 to September 2021, regardless of sex, from patients who underwent colonoscopies in the Digestive Service of Health Area IV of Asturias (Spain) as part of CRC screening. Most had a positive fecal occult blood test (64.4%). All the colonoscopies were performed before noon by the same doctor. The inclusion criterion was a score of 1 to 2 on the American Society of Anesthesiologists classification [16]. Some patients presented more than one lesion, even with different classifications in pathological anatomy. For classification purposes, these patients were grouped according to the lesions in the most malignant category.

At the time of recruitment, the likelihood of colorectal lesions in participants was unknown. The objective of the research was explained to the participants, who consented to participate. The participants collected a recent fecal sample at home in a sterile sample collection container and kept it refrigerated constantly. Subsequently, aliquots of approximately 200 mg were introduced in sterile Eppendorf tubes to be preserved at −80 °C until the time of their use. In addition, the majority of participants (53 out of 59) completed a nutritional survey.

The study considered several variables, such as age, body mass index (BMI), sex, polyamines, and other food components of the diet, along with the content of amines in feces, which was determined by high-performance liquid chromatography (HPLC). In addition, the presence or absence of colorectal lesions was examined as determined by colonoscopy and classified as a dichotomic variable. The colorectal lesions were later subdivided based on anatomical pathology diagnosis.

The research was approved by the Comité de Ética de la Investigación del Principado de Asturias (Spain) (CElmPA 28/19, approved on 5 March 2019) and adhered to the guidelines of the Declaration of Helsinki.

### 2.2. Daily Intake of Food and Polyamines

Nutritional surveys were conducted with the participants using the VioScreen food frequency questionnaire (FFQ) (Viocare Inc., Princeton, NJ, USA), which had already been previously used on the same population and provides information on intake for up to 90 days [5]. This survey took approximately 20 min to complete, had optimal reliability and validity [17], and was conducted by one trained interviewer on all the participants. The survey also reported Healthy Eating Index (HEI) and the Dietary Inflammatory Index (DII) scores. The HEI score ranges from 0 to 100, with higher scores indicating better diet quality. Scores > 80 reflect a good diet, a median score from 51 to 80 shows that the diet needs improvement, and scores < 51 indicate a poor diet [18]. The DII score ranges from negative to positive values, usually from −5.5 to 5.5, with greater negativity associated with more anti-inflammatory diets and greater positivity with pro-inflammatory properties [4]. Additionally, the Prevención con Dieta Mediterránea (PREDIMED) survey, consisting of 14 questions, estimated adherence to the Mediterranean diet. The score categories include high adherence (12–14), moderate adherence (8.0–11.99), low adherence (5.0–7.99), and very low adherence (<5.0) [19].

Polyamine consumption was determined using a previously elaborated database on the composition of putrescine, spermidine, and spermine in mg per 100 g or ml of raw foods [5]. The polyamine intake of putrescine, spermidine, and spermine was expressed in mg of daily ingestion per person. The sum of the three polyamines yielded the total polyamine intake, which was also described as µmol per person per day. The ratios of ingested polyamines were also determined.

### 2.3. Determination of Polyamines in Feces via High-Performance Liquid Chromatography (HPLC)

The amines were determined using a pre-column derivatization method. The samples of feces were homogenized in added purified water (weight of the sample × 10) and then centrifuged at 4000 rpm for 3 min at room temperature to obtain 300 µL of supernatant. Next, the samples were treated with perchloric acid for 10 min at 4 °C to produce a final concentration of 15.8% and were processed and chromatographed by HPLC (Shimadzu Prominence) as previously described [20]. The determinations were expressed as nmol/mg of feces. The ratios of fecal polyamines were also calculated.

The compounds used in this research were purchased from Sigma-Aldrich (Madrid, Spain), including putrescine (tetramethylenediamine), spermidine (*N*-[3-aminopropyl]-1,4-butanediamine), spermine (*N*,*N*′-bis[3-aminopropyl]-1,4-butanediamine), *N*-acetylputrescine hydrochloride, *N*-acetylspermidine hydrochloride, isoamylamine (isopentylamine: 1-amino-3-methylbutane), cadaverine (cadaverine dihydrochloride), tyramine (2-[4-hydroxyphenyl] ethylamine), tyramine hydrochloride (4-[2-aminoethyl] phenol hydrochloride), and 2-hydroxydiaminopropane. The drugs were dissolved in purified water with a resistivity of 10–15 MΩ·cm.

### 2.4. Statistical Analyses

As most variables did not follow a normal distribution (based on the Shapiro–Wilk test and homogeneity of variance), nonparametric statistics were used to express the data (median and 25th–75th percentiles) and to compare the differences between various factors. Box-and-whisker plots were used to illustrate the content of *N*-acetyl putrescine and cadaverine in feces. To calculate statistical differences, the Mann–Whitney U-test was used to compare two independent groups (*p*-values were adjusted by Bonferroni correction), and the Kruskal–Wallis test (pairwise comparison *p*-values adjusted by the Bonferroni correction for multiple tests) was used to compare multiple groups.

Pearson’s correlation coefficient (*r*) (two-tailed) was used to determine the strength of the association between variables, classified as weak (up to 0.3), moderate (from ~0.4 to 0.6), or strong (from ~0.7 to 0.9). Multiple regression analysis was used to predict fecal polyamines as dependent variables based on ingested dietary components. Binary logistic regression was used to predict the absence or presence of colorectal lesions in the subjects studied. Discriminant function analysis was undertaken to determine the difference between subjects without lesions, those with benign conditions (hyperplastic lesions), those with precancerous conditions (tubular and tubulovillous adenomas), and subjects with malignant conditions (adenocarcinomas).

For all analyses, values of *p* ≤ 0.05 were considered significant. The analyses were performed using IBM SPSS Statistics version 27.0 (IBM Corp., Armonk, NY, USA).

## 3. Results

### 3.1. Study Participants and Types of Colorectal Lesion

A total of 59 participants took part in the study, of whom 37 (62.7%) were male and 22 (37.3%) female. Among the participants, 18 (30.5%) had no colorectal lesions, whereas 41 (69.5%) did have lesions. Analysis of the anatomical pathology of tissue samples taken from the colon and rectum revealed that some patients had more than one type of lesion, including lesions of different pathological anatomies. Most patients had from 1 to 5 lesions (87.8%). The lesions were located mainly in the sigmoid colon (44.34%), and tubular adenomas were the most prevalent (65.1%) (Table 1). Patients with multiple lesions were assigned to the most malignant category. The study revealed that 6 patients had only hyperplastic lesions (14.6%), 26 had tubular adenomas (63.4%), 6 had tubulovillous adenomas (14.6%), and 3 had adenocarcinomas (7.3%).

The participants’ ages ranged from 40 to 70 years, with the median age being 61. The median age of males was 58 and that of females was 61. The Mann–Whitney U-test identified no significant differences in age and BMI between the participants with and those without colorectal lesions (Table 2).

### 3.2. Association of Nutritional Survey Data and the Presence of Colorectal Lesions

First, the data regarding the absence or presence of colorectal lesions were analyzed. The PREDIMED survey showed that 63.6% of subjects without colorectal lesions had moderate (45.5%) to high adherence (18.2%) to the Mediterranean diet, whereas 46.2% of those with colorectal lesions had moderate (38.5%) to high adherence (7.7%).

Regarding the subjects’ diet (estimated via the FFQ), no significant differences were found in median caloric intake between the subjects with and those without colorectal lesions. The overall median HEI score was 72.3, ranging from 49.6 to 89.8, being 75.8 (57.8–85.7) in patients without lesions, and 68.4 (49.6–89.9) in those with colorectal lesions. The overall median DII score was −1.6 (range: −4.75 to 2.15); the median score in the patients without lesions was −2.09 (−4.75 to 1.47), and it was −1.32 (−4.54 to 2.15) in those with colorectal lesions. The differences in HEI and DII scores found between patients were not significant (Table 2).

The PREDIMED score for adherence to the Mediterranean diet had a significant moderate positive correlation with the HEI score (*r* = 0.498, *p* = 0.002) and a negative one with the DII score (*r* = −0.619, *p* < 0.001). A strong significant inverse correlation was found between the HEI and DII scores (*r* = −0.762, *p* < 0.001).

The FFQ revealed significant differences in dietary components between patients without and with colorectal lesions, as shown by Mann–Whitney analysis. Patients with colorectal lesions had a significantly higher intake of alcohol servings (and the corresponding calories), beta-cryptoxanthin, number of citruses, melon, or berry cup equivalents, eggs, galactose, glucose, inositol, and lignan secoisolariciresinol, and they had lower intake of low-fat dairy servings compared with those without colorectal lesions (Table 2).

The Kruskal–Wallis analysis comparing the intake of dietary components between three groups of patients, those without colorectal lesions, those with benign lesions, and those with precancerous and malignant colorectal lesions (as diagnosed by anatomical pathology), revealed significant differences between the groups in the following categories: daily alcohol servings (and corresponding calories), cholesterol, fish servings and non-fried fish servings, other seafood low in omega-3, fructose, glucose, inositol, lignan secoisolariciresinol, cooked lean meat, poultry, some types of monounsaturated fatty acids, polyunsaturated fatty acids, saturated fatty acids, and niacin. Pairwise comparison among the groups, adjusted by the Bonferroni correction, revealed differences in some dietary components between the subjects without and with colorectal lesions (Table 2).

### 3.3. Association of Dietary Intake of Polyamines with Colorectal Lesions in Patients

The median values of daily polyamine intake per person showed that patients with colorectal lesions consumed significantly more putrescine in the diet than subjects without colorectal lesions (*p* = 0.026). No differences were found in the intake of spermidine and spermine or in the total daily intake of polyamines. The analysis of the ratios of ingested polyamines identified significantly higher putrescine–spermidine ratios (*p* = 0.008) and putrescine–spermine ratios (*p* = 0.042) in patients with colorectal lesions than in those without lesions (Table 3).

The Kruskal–Wallis analysis showed differences between the groups in the ratio of ingested putrescine–spermidine (*p* = 0.031). After adjusting for multiple comparisons, the ratio was significantly higher in patients with precancerous and malignant/cancerous lesions than in those without lesions (*p* = 0.032) (Table 3).

### 3.4. Association of Biogenic Amines in Feces with Colorectal Lesions in Patients

**Figure 1:**
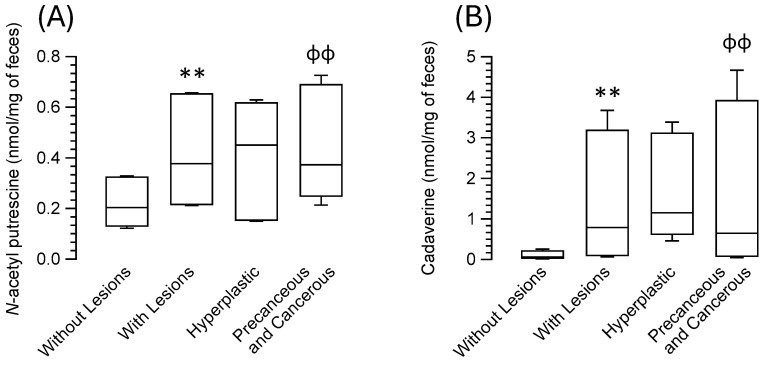
Box- and-whisker plots of feces content of (**A**) *N*-acetyl putrescine and (**B**) cadaverine, comparing groups of patients: those without colorectal lesions with all patients with colorectal lesions, and separately as hyperplastic lesions and precancerous and cancerous lesions. ** *p* ≤ 0.01 patients with lesions compared with those without lesions, using the Mann-Whitney test; ^ɸɸ^ *p* ≤ 0.01 comparing the patients without lesions with those with hyperplastic lesions and precancerous and cancerous lesions, using the Kruskal–Wallis test adjusted by Bonferroni multiple comparisons.

The analysis of the content of biogenic amines in the feces, based on the absence or presence of colorectal lesions, revealed that patients with colorectal lesions had significantly higher levels of *N*-acetyl putrescine (Figure 1A) (*p* = 0.0015) and cadaverine (Figure 1B) (*p* = 0.0014) than those without colorectal lesions. No significant differences were observed for the rest of the amines. However, differences did exist in the fecal content ratios of putrescine–cadaverine (*p* = 0.024), *N*-acetyl putrescine–cadaverine (*p* = 0.009), and cadaverine–tyramine (*p* < 0.001) (Table 3).

The Kruskal–Wallis analysis of biogenic amines in the feces of patients without colorectal lesions, with benign lesions, and with precancerous and malignant lesions revealed differences between the three groups in terms of the presence of *N*-acetyl putrescine (Figure 1A) (*p* = 0.006) and cadaverine (Figure 1B) (*p* = 0.006) and in the ratios of putrescine–cadaverine (*p* = 0.048), *N*-acetyl putrescine–cadaverine (*p* = 0.021), and cadaverine–tyramine (*p* = 0.002) (Table 2). The pairwise comparison (with Bonferroni correction) showed that *N*-acetyl putrescine (*p* = 0.005) and cadaverine (*p* = 0.007) were significantly higher in patients with precancerous lesions and adenocarcinomas than in patients without lesions and that the cadaverine–tyramine ratio in patients with benign lesions differed significantly from that in patients with precancerous or malignant/cancerous colorectal lesions (Table 3). No differences were seen regarding the colorectal location of those lesions.

The separate analyses of linear correlation in subjects without and with colorectal lesions revealed a moderately significant positive correlation in those without lesions between feces putrescine and spermidine (*r* = 0.685, *p* = 0.002, *n* = 18), *N*-acetyl putrescine (*r* = 0.606, *p* = 0.008, *n* = 18), and cadaverine (*r* = 0.591, *p* = 0.01, *n* = 18). Spermidine strongly correlated positively with spermine (*r* = 0.873, *p* < 0.001, *n* = 18), *N*-acetyl putrescine (*r* = 0.9, *p* < 0.001, *n* = 18), and *N*-acetyl spermidine (*r* = 0.699, *p* = 0.011, *n* = 12). *N*-acetyl putrescine strongly correlated positively with *N*-acetyl spermidine (*r* = 0.745, *p* = 0.005, *n* = 12) but not with cadaverine.

In patients with colorectal lesions, putrescine moderately positively and significantly correlated with *N*-acetyl putrescine (*r* = 0.582, *p* < 0.001, *n* = 41), cadaverine (*r* = 0.525, *p* = 0, *n* = 41), and tyramine (*r* = 0.74, *p* = 0, *n* = 40). Spermidine moderately correlated positively and significantly with spermine (*r* = 0.408, *p* = 0.008, *n* = 41), and spermine correlated positively and significantly with *N*-acetyl spermidine (*r* = 0.452, *p* = 0.008, *n* = 41) and cadaverine (*r* = 0.569, *p* = 0.008, *n* = 33). Finally, *N*-acetyl putrescine correlated positively with cadaverine (*r* = 0.444, *p* < 0.001, *n* = 41).

When lesions were analyzed for malignity, fecal spermidine strongly and positively correlated with spermine (*r* = 0.865, *p* = 0.026, *n* = 6) and tyramine (*r* = 0.890, *p* = 0.017, *n* = 35) in patients with hyperplastic lesions. In patients with precancerous and adenocarcinomas lesions, putrescine moderately correlated with *N*-acetyl putrescine (*r* = 0.579, *p* < 0.001, *n* = 35) and cadaverine (*r* = 0.522, *p* < 0.001, *n* = 35) and strongly with tyramine (*r* = 0.740, *p* < 0.001, *n* = 35). Spermidine weakly correlated with spermine (*r* = 0.391, *p* = 0.02, *n* = 35), spermine moderately correlated with *N*-acetyl spermidine (*r* = 0.456, *p* = 0.017, *n* = 35) and cadaverine (*r* = 0.611, *p* < 0.001, *n* = 35), and *N*-acetyl putrescine correlated with cadaverine (*r* = 0.436, *p* = 0.009, *n* = 35).

### 3.5. Correlation between Dietary Intake of Polyamines and Their Fecal Content in Relation to Colorectal Lesion Types

In subjects without lesions, putrescine intake moderately correlated positively with fecal *N*-acetyl putrescine (*r* = 0.536, *p* = 0.033, *n* = 16) and strongly with *N*-acetyl spermidine (*r* = 0.717, *p* = 0.02, *n* = 6). In patients with benign lesions, putrescine intake strongly correlated with fecal putrescine (*r* = 0.917, *p* = 0.01, *n* = 6) and cadaverine (*r* = 0.87, *p* = 0.026, *n* = 6), and dietary spermine correlated with fecal cadaverine (*r* = 0.862, *p* = 0.027, *n* = 6). Moreover, in patients with malignant lesions, spermidine intake moderately correlated with fecal spermine (*r* = 0.502, *p* = 0.004, *n* = 31), and spermine intake weakly correlated with fecal cadaverine (*r* = 0.398, *p* = 0.027, *n* = 31). Finally, the putrescine–spermidine intake ratio was moderately inversely correlated with fecal cadaverine content (*r* = −0.402, *p* = 0.025, *n* = 31).

### 3.6. Discriminant Function Analysis of Subjects without Lesions, with Benign Lesions, and with Precancerous or Malignant Lesions Based on Polyamine Intake and Fecal Content of N-acetyl Putrescine and Cadaverine

**Figure 2:**
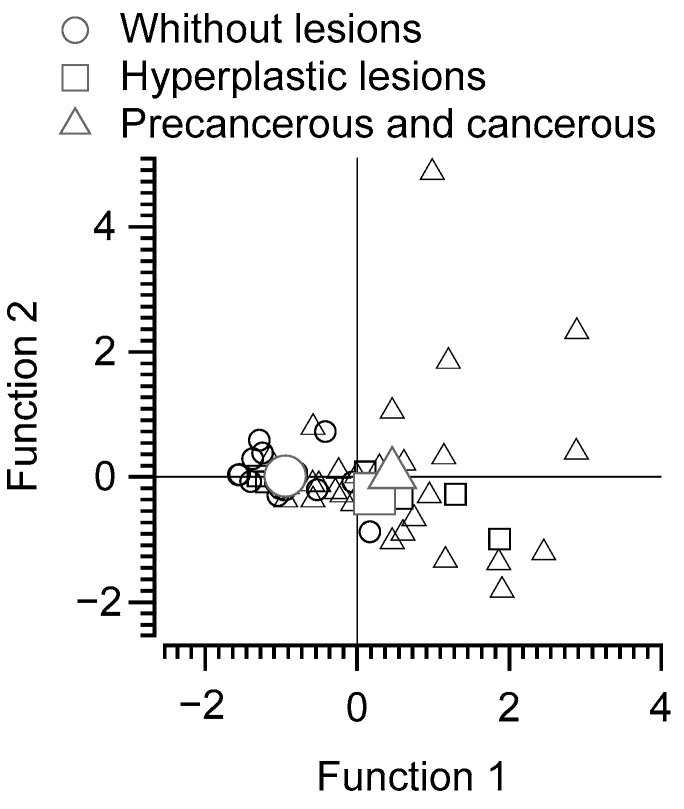
Discriminant function analysis of subjects without colorectal, hyperplastic and precancerous and cancerous lesions. Cadaverine content in feces and the ratio of putrescine/spermidine dietary intake fits the first function, and tyramine and *N*-acetyl putrescine the second function.

The discriminant analysis of the ratio of putrescine–spermidine intake to fecal content of *N*-acetyl putrescine, cadaverine, and tyramine revealed two discriminant functions. The first explained 97.7% of the variance (canonical *R*^2^ = 0.3), whereas the second explained only 2.3% (canonical *R*^2^ = 0.01). In combination, these discriminant functions significantly differentiated the patient groups (Ʌ = 0.69, χ^2^[8] = 17.37, *p* = 0.027), but removing the first function indicated that the second function did not significantly differentiate the treatment groups (Ʌ = 0.99, χ^2^[3] = 0.466, *p* = 0.926). The correlations between the outcomes and the discriminant functions revealed that cadaverine content in feces and the ratio of putrescine/spermidine dietary intake correlated highly and fairly evenly onto the first function (*r* = 0.60 and 0.57, respectively) but less onto the second (*r* = 0.46 and −0.50, respectively); tyramine and N-acetyl putrescine in feces correlated more highly onto the second function (*r* = 0.72 and *r* = 0.62, respectively) than onto the first function (*r* = 0.10 and *r* = 0.44, respectively). The classification results yielded a prediction of 75% for patients without colorectal lesions, 86.7% for patients with precancerous and cancerous lesions, and 0% for those with hyperplastic lesions. The discriminant function plot shows that the first function differentiated the group of patients with malignant and hyperplastic lesions from those without colorectal lesions, whereas the second function was less discriminant (Figure 2).

### 3.7. Multiple Regression Analysis on Fecal Polyamines as Dependent Variables

This analysis was undertaken to predict the fecal content of the polyamines that increased in patients with colorectal lesions (*N*-acetyl putrescine and cadaverine), based on dietary components, including polyamines, as independent variables. The results showed that *N*-acetyl putrescine content could be predicted in 41% of cases by daily ingestion of whole grain servings and lignan secoisolariciresinol in addition to the corresponding constant. The wholegrain servings were able to predict cadaverine content in 10% of cases (Table 4).

### 3.8. Logistic Regression Analysis to Predict the Presence of Colorectal Lesions

A logistic regression analysis was performed to determine the predictive value of dietary polyamines and their content in feces for the existence of colorectal lesions. The results showed that the content of cadaverine (*p* = 0.018) and tyramine (*p* = 0.032) in feces, the ratio of dietary intake of putrescine–spermidine (*p* = 0.017), and a constant (*p* = 0.013) together predicted the presence or absence of colorectal lesions in 84.6% of subjects. Specifically, the analysis predicted the absence of lesions in 81.3% of the subjects and the presence of lesions in 86.11% of the patients. The equation derived from the analysis was Y(Without lesions/With lesions) = −3.504 + (6.204 × cadaverine in nmol/mg of feces) + (−20.877 × tyramine in nmol/mg of feces) + (1.862 × ratio of dietary intake of putrescine/spermidine). The cutoff value for the absence or presence of lesions was 0.5.

## 4. Discussion

The present study found that patients with colorectal lesions tended to have higher levels of *N*-acetyl putrescine and cadaverine in their feces, which may suggest changes in their microbiota. This study also found that patients with colorectal lesions tended to consume diets richer in putrescine and with a higher putrescine–spermidine ratio.

Our findings, in line with those of other studies, indicate that patients with lower adherence to the Mediterranean diet [21] (estimated by PREDIMED) [19], with less healthy diets [22] (by HEI score) [18], and with lower anti-inflammatory capabilities [23] (DII score [4]) were more likely to be diagnosed with colorectal lesions. These well-established associations underscore the importance of healthy and anti-inflammatory diets in preventing CRC. A Western diet, which typically includes more foods from animal products, has been linked to a higher incidence of CRC compared with the Mediterranean diet, which emphasizes fruits and vegetables [24].

This study identified an increase in specific dietary components in patients with colorectal lesions. Patients with those lesions tended to consume greater amounts of alcohol, eggs, galactose, and glucose and had a lower intake of low-fat dairy. These factors have been linked to the pathogenesis of precancerous lesions and CRC due to their direct effect on intestinal epithelial cells, the compounds produced after metabolization, or their impact on the microbiota [9,10]. However, patients with those lesions also consumed greater amounts than healthy subjects of dietary components that protect against CRC, such as inositol, citrus, melon, berry, and lignan secoisolariciresinol. The involvement of inositol and lignan secoisolariciresinol is controversial. Overall, the intake of dietary components was unequally affected depending on the malignity of the lesions in our study.

Polyamines are crucial for developing and maturing the gastrointestinal tract and establishing the microbiota and the immune system from birth [25,26]. The intestinal epithelium lacks blood vessels and is nourished primarily by polyamines from the intestinal lumen in addition to those from intracellular synthesis. In the upper parts of the small intestine, where they are absorbed, polyamines come mainly from the diet; in the colon, they come mainly from polyamine-producing microbiota [27,28]. Their luminal presence is essential throughout life to preserve the physical and functional integrity of the gastrointestinal tract [28]. Polyamines have also been linked to the development of CRC, which is why difluoromethylornithine (eflornithine), an inhibitor of polyamine synthesis, is used in the chemoprevention of CRC [8].

It is valuable to determine the dietary intake of polyamines in order to analyze their association with fecal polyamines and colorectal lesions. This area of research has been limited to a few studies [29,30,31], as the intake of polyamines in populations is not regularly assessed. We used previously compiled tables to calculate the participants’ intake of polyamines [5]. The median values in subjects without colorectal lesions were similar to those reported in the general population [5]. In patients with colorectal lesions, however, the dietary intake of putrescine was significantly higher; more specifically, increased putrescine–spermidine and putrescine–spermine ratios were more likely to be associated with colorectal lesions, and the daily dietary putrescine–spermidine ratio was more likely to be associated with malignant lesions. This finding underscores the potential implications of maintaining an adequate spermidine intake in relation to putrescine. This aligns with the reported association of spermidine with decreased intestinal inflammatory pathologies and its chemoprophylactic action against CRC [32]. Previous studies have reported contradictory findings regarding whether dietary polyamine consumption should be considered a risk [30] or a protective factor [29,31].

One interesting finding is the discovery of changes in the relationships between dietary polyamines and their presence in the feces of patients with colorectal lesions, especially those with malignant lesions. In those patients, the intake of dietary putrescine was found to be positively correlated with levels of *N*-acetyl putrescine and cadaverine in the feces. Additionally, the putrescine–spermine intake ratio was inversely correlated with fecal cadaverine levels. These associations suggest that foods containing putrescine could lead to byproducts that affect the gut microbiota, resulting in metabolite changes [3,33]. Dietary putrescine is unlikely to reach the colorectal tract and be converted to other amines, as it is absorbed in the upper part of the small intestine [34].

The microbiota is the primary source of polyamines in the large intestine. These metabolites play a role in maintaining the colorectal tract’s physiology and in carcinogenesis [10]. Monitoring levels of polyamines can help to identify imbalances in the gut microbiota, which dietary factors can influence [3,9]. Our research confirms previous findings that putrescine and spermidine are common polyamines in healthy individuals’ feces, followed by cadaverine and spermine, although isoamylamine and *N*-acetyl putrescine content were the most prevalent amines. Changes occurred in the correlation between polyamines in feces when patients presented colorectal lesions along with a significant increase in *N*-acetyl putrescine and cadaverine, especially in patients with precancerous and cancerous lesions, compared with those without lesions. These fecal changes may have resulted from dysbiosis of the fecal microbiota due to bacteria linked to human CRC [11,12,13].

*N*-acetyl putrescine and cadaverine content in feces correlated positively, at least partially, suggesting a common source. Among other bacteria, certain Gram-negative ones, such as Escherichia coli and some Enterococcus strains, have been found to produce cadaverine [35]. Furthermore, *Escherichia coli* also produces *N*-acetyl putrescine [35]. Other studies have also reported an increase in fecal cadaverine and putrescine linked to colorectal tumorigenesis [36]. However, putrescine did not increase in our study’s patients. According to our results, *N*-acetyl putrescine and cadaverine fecal content could help identify colorectal lesions in 20% of patients. These microbial metabolites could also complement existing noninvasive tests and assist in the early detection of colorectal lesions. Additionally, there have been proposals to diagnose CRC through the measurement of polyamines in plasma [37], urine [38], and saliva [39]. Taking into account the putrescine–spermidine intake ratio and the fecal content of *N*-acetyl putrescine, cadaverine, and tyramine, the discriminant function analysis differentiated patients with malignant colorectal lesions from healthy subjects.

An individual’s dietary patterns may influence polyamine-producing bacteria [3,9,10]. The linear regression analysis, including the corresponding constant, showed that the intake of wholegrain servings and lignan secoisolariciresinol could predict a 41% variance of *N*-acetyl putrescine. In comparison, the intake of wholegrain servings predicted only a 10% variance in cadaverine, suggesting different colorectal sources of these polyamines. In addition to effects on cellular tropism, biogenic amines (metabolites of the microbiota) modulate the peristalsis of intestinal tissues and segmentation contractions [7]. They can lead to intestinal symptoms in CRC patients, such as changes in stool frequency and consistency as well as constipation or diarrhea. Traced amine-associated receptors may trigger these effects when exposed to diverse gut biogenic amines, reflecting a putative link between nutrient intake, the microbiota, and the gastrointestinal tract [40].

## 5. Conclusions

The present study suggests the importance of determining polyamine content in the diet, especially the putrescine–spermidine ratio, as a potential factor affecting an imbalance associated with colorectal lesions. Additionally, according to our results, detection of *N*-acetyl putrescine and cadaverine in feces could be helpful as biological markers of colorectal lesions, potentially predicting with a given level of accuracy the presence or absence of lesions, without considering gender differences.

It is important to note the limitations of this study, especially its relevance to broader populations. The non-random selection of the sample may have introduced biases. However, it is worth mentioning that the participants were recruited consecutively based on the inclusion criteria through the public health system that serves a significant portion of the population in the corresponding geographical area, regardless of their socioeconomic and cultural diversity.

## Figures and Tables

**Table 1:** Number of colorectal lesions per patient, their location, and classification according to anatomical pathology, as well as the patients presenting them, in absolute values and as percentages of the total occurrences.

**Number of Colorectal Lesions**	**Number of Patients**	**Percentage**
1	14	34.15
2	11	26.83
3–5	11	26.83
6–10	5	12.20
**Location of Colorectal Lesions**	**Number of Lesions**	**Percentage**
Ascending colon	20	18.87
Transverse colon	11	10.38
Descending colon	9	8.49
Sigmoid colon	47	44.34
Rectum	19	17.92
**Anatomical Pathology Classification**	**Number of Lesions**	**Percentage**
Hyperplastic lesions	23	21.70
Tubular adenomas	69	65.09
Tubulovillous adenomas	11	10.38
Adenocarcinomas	3	2.83

**Table 2:** Age, nutritional characteristics, and daily dietary components for the participants included in the study, expressed as the median (25th–75th percentiles).

Variable	Without Colorectal Lesions	With Colorectal Lesions	Hyperplastic Lesions	Precancerous and Adenocarcinomas
Characteristics of participants and of diets				
Age (years)	58.42 (52.23–65.41)	61.25 (56.24–64.93)	58.73 (54.86–61.16)	62.17 (56.44–65.22)
Calories (kcal/day)	1973.83 (1642.46–2162.3)	2120.13 (1909.81–2618.88)	2918.25 (1923.8–3070.51)	2108.4 (1907.34–2470.14)
BMI (kg/m^2^)	26.45 (22.78–34.23)	27 (25–29.7)	24.85 (24.05–34.05)	27.1 (25.4–29.4)
HEI Score	75.8 (67.05–80.23)	68.4 (61.85–79.65)	65.7 (56.25–79.4)	72.1 (61.9–80)
DII Score	−2.09 (−3.4–−0.44)	−1.32 (−3.01–0.59)	−1.1 (−3.48–1.15)	−1.32 (−2.9–−0.19)
Components of daily diet				
Alcohol calories (kcal)	26.82 (13.84–39.71)	105.92 (20.13–260.43) *	228.7 (9.62–362.92)	105.02 (20.76–253.83)
Alcohol servings	0.2 (0.11–0.28)	0.85 (0.17–2.19) **	1.93 (0.1–2.84)	0.73 (0.18–2.16) ^ɸ^
Cholesterol (mg)	211.22 (150.75–303.79)	296.86 (183.75–341.12)	365.47 (288.64–453.69) ^ɸ^	265.89 (167.32–337.76)
Number of citrus, melon, berry cup equivalents (cups)	0.56 (0.18–0.64)	0.77 (0.3–1.36) *	0.93 (0.3–2.69)	0.77 (0.27–1.3)
Fish servings	1.92 (0.87–2.94)	2.21 (1.31–3.43)	4.64 (2.19–7.79)	1.7 (1.16–3.08)
Fructose (g)	18.22 (14.97–22.41)	22.36 (17.74–28.15)	27.94 (23.39–34.54) ^ɸ^	20.68 (17.09–25.75)
Galactose (g)	0.26 (0.13–0.47)	0.48 (0.21–1.96) *	1.22 (0.23–2.12)	0.42 (0.21–1.95)
Glucose (g)	16.71 (15.12–18.49)	21.17 (16.09–27.94) *	28.64 (23.74–32.92) ^ɸɸ^	20.44 (15.26–24.7)
Inositol (g)	0.42 (0.33–0.52)	0.56 (0.4–0.85) *	0.65 (0.39–1.33)	0.56 (0.4–0.83)
Lignan secoisolariciresinol	65.21 (54.38–102.47)	103.97 (65.44–139.35) *	132.23 (90.22–151.01)	91.99 (59.79–139.95)
Low-fat dairy servings	1.62 (0.49–2.41)	0.53 (0.06–1.5) *	0.64 (0.2–0.98)	0.45 (0.05–1.5)
Eggs (equivalent to 28.3 g of lean meat)	0.38 (0.29–0.63)	0.63 (0.41–1.04) *	1.05 (0.52–1.11)	0.6 (0.41–0.86)
Cooked lean meat from fish, other seafood low in omega-3 (28.3 g)	0.78 (0.31–1.06)	0.84 (0.42–1.21)	1.6 (0.94–2.62) ^¥^	0.63 (0.35–1.08)
Cooked lean meat from meat, poultry, fish (28.3 g)	3.36 (2.15–5.47)	3.38 (2.47–4.88)	5.57 (4.27–6.98) ^¥^	3 (2.38–4.3)
MUFA 16:1 (palmitoleic acid) (g)	0.86 (0.54–1.07)	0.98 (0.75–1.23)	1.36 (0.97–1.57) ^ɸ^	0.94 (0.72–1.2)
MUFA 20:1 (gadoleic acid) (g)	0.2 (0.16–0.28)	0.22 (0.18–0.32)	0.35 (0.25–0.49) ^ɸ^	0.21 (0.17–0.27)
Niacin equivalents (mg)	39.02 (29.09–51.83)	39.14 (33.86–47.22)	51.12 (43.27–57.46) ^¥^	38.08 (33.29–45.98)
Non-fried fish servings	1.31 (0.59–2.58)	2.08 (0.99–3.32)	4.35 (2–6.46) ^ɸ^	1.7 (0.87–2.81)
PUFA 18:3 n-6 (g)	0.02 (0.01–0.02)	0.02 (0.01–0.03)	0.03 (0.02–0.04) ^¥^	0.01 (0.01–0.02)
PUFA 20:4 (arachidonic acid) (g)	0.11 (0.06–0.14)	0.12 (0.09–0.16)	0.17 (0.14–0.21) ^ɸ^	0.11 (0.08–0.16)
SFA 17:0 (margaric acid) (g)	0.08 (0.06–0.12)	0.1 (0.07–0.14)	0.16 (0.1–0.21) ^ɸ^	0.1 (0.06–0.12)
SFA 4:0 (butyric acid) (g)	0.28 (0.12–0.43)	0.4 (0.23–0.74)	0.66 (0.33–0.99) ^ɸ^	0.37 (0.19–0.69)

* *p* ≤ 0.05 and ** *p* ≤ 0.01 by *t*-test comparison between subjects without and with colorectal lesions, Mann–Whitney U test; ^ɸ^ *p* ≤ 0.05 and ^ɸɸ^ *p* ≤ 0.01 vs. patients without colorectal lesions and ^¥^ *p* ≤ 0.05 patients with hyperplastic lesions vs. patients with precancerous lesions and adenocarcinomas, using the Kruskal–Wallis test adjusted with Bonferroni multiple comparisons.

**Table 3:** Dietary intake of polyamines in mg per person and day, their ratios, and intake in mg per kcal, as well as the content of polyamines in feces in nmol per mg of sample, expressed as median (25th–75th percentiles).

Variable	Without Colorectal Lesions	With Colorectal Lesions	Hyperplastic Lesions	Precancerous and Adenocarcinomas
Dietary intake of polyamines in mg per person and day				
Putrescine	15.58 (11.11–20.66)	23.81 (12.6–33.88) *	27.51 (14.52–35.63)	22.17 (11.88–34.09)
Spermidine	10.93 (8.1–14.4)	11.22 (8.47–12.5)	11.24 (10.16–14.24)	11.19 (8.05–12.5)
Spermine	5.98 (4.2–11.45)	7.35 (5.47–9.01)	8.89 (7.96–12.91)	7.09 (4.98–8.5)
Total polyamines	33.37 (24.71–46.66)	42.91 (28.09–50.61)	46.88 (35.56–58.25)	42.19 (27.36–50.61)
Ratios of dietary intake of polyamines				
Putrescine–spermidine ratio	1.46 (0.97–1.85)	2.12 (1.46–3.06) **	2.47 (1.18–3.04)	2.01 (1.47–3.12) ^ɸ^
Putrescine–spermine ratio	1.91 (1.41–3.14)	3.17 (2.13–4.28) *	2.71 (1.66–3.47)	3.4 (2.15–4.33)
Spermidine–spermine ratio	1.56 (1.24–1.86)	1.44 (1.31–1.81)	1.3 (1.01–1.47)	1.48 (1.35–1.86)
Dietary intake of polyamines in mg per kcal per person and day				
Putrescine	8.25 (5.34–10.48)	10.46 (5.96–14.95)	9.43 (5.33–15.99)	10.49 (6.13–14.12)
Spermidine	5.76 (4.38–7.19)	5.01 (3.87–6.01)	4.96 (3.55–5.95)	5.01 (3.9–6.06)
Spermine	3.83 (2.22–5.25)	3.29 (2.81–4.19)	3.88 (2.86–4.84)	3.21 (2.73–4.18)
Total polyamines	18.73 (12.18–22.65)	18.27 (14.09–25.87)	16.16 (14.77–26.6)	19.06 (13.74–25.35)
Feces polyamines in nmol per mg of sample				
Putrescine	0.55 (0.21–1.02)	0.64 (0.38–1.95)	0.91 (0.33–2.62)	0.58 (0.35–1.9)
Spermidine	0.61 (0.43–0.94)	0.99 (0.55–1.43)	1.15 (0.56–1.51)	0.87 (0.53–1.4)
Spermine	0.03 (0.01–0.04)	0.03 (0.02–0.05)	0.04 (0.03–0.07)	0.03 (0.02–0.05)
*N*-acetyl putrescine	0.89 (0.56–1.39)	1.58 (0.92–2.7) ***	1.88 (0.67–2.59)	1.56 (0.93–2.98) ^ɸɸ^
*N*-acetyl spermidine	0.17 (0.11–0.22)	0.29 (0.16–0.43)	0.27 (0.14–0.54)	0.29 (0.16–0.43)
Cadaverine	0.24 (0.06–1.02)	3.14 (0.25–14.71) ***	4.6 (1.83–13.55)	2.58 (0.18–18.66) ^ɸɸ^
Tyramine	0.08 (0.06–0.13)	0.09 (0.06–0.16)	0.1 (0.07–0.13)	0.08 (0.06–0.21)
Isoamylamine	2.04 (1.37–2.72)	2.1 (0.38–2.74)	1.51 (0.18–2.76)	2.1 (0.77–2.81)
Ratios of feces content of polyamines				
Putrescine–spermidine ratio	0.8 (0.36–1.41)	0.93 (0.32–2.67)	0.8 (0.27–2.65)	1.08 (0.35–2.87)
Putrescine–spermine ratio	19.56 (10.47–32.67)	22.45 (7.89–50.18)	23.35 (5.14–67.38)	22.45 (7.92–55.05)
Spermidine–spermine ratio	25.53 (17.99–35.76)	22.85 (16.46–35.87)	23.34 (18.64–31.82)	22.85 (16.11–38.78)
Putrescine–cadaverine ratio	1.77 (0.45–9.22)	0.52 (0.1–1.47) *	0.19 (0.07–2.76)	0.67 (0.13–1.55)
Putrescine–*N*-acetyl putrescine ratio	0.55 (0.28–0.85)	0.6 (0.23–1.22)	0.78 (0.14–1.34)	0.53 (0.24–1.23)
*N*-acetyl putrescine–cadaverine ratio	2.88 (0.79–14.67)	0.7 (0.21–5.19) **	0.33 (0.15–2.59)	0.79 (0.21–5.2) ^¥^
Cadaverine–tyramine ratio	2.31 (0.92–11.23)	20.04 (3.67–112.14) ***	48.8 (27.41–153.41) ^ɸ^	14.26 (3.55–111.52) ^ɸɸ^

* *p* ≤ 0.05, ** *p* ≤ 0.01, and *** *p* ≤ 0.001 by *t*-test comparison between subjects without and with colorectal lesions, using the Mann–Whitney U test; ^ɸ^ *p* ≤ 0.05 and ^ɸɸ^ *p* ≤ 0.01 vs. patients without colorectal lesions and ^¥^ *p* ≤ 0.05 patients with hyperplastic lesions vs. patients with precancerous lesions and adenocarcinomas, using the Kruskal–Wallis test adjusted by Bonferroni multiple comparisons.

**Table 4:** Linear regression analysis results for the predictive fecal content of *N*-acetyl putrescine, two models, and cadaverine, based on specific diet components (independent variables).

Regression Model Variables for Each Amine	B	SE B	β	*p*
*N*-acetyl putrescine in feces (*R*^2^ = 0.41, ANOVA *p* < 0.001)				
Constant	0.579	0.101		<0.001
Wholegrain servings	0.177	0.030	0.709	<0.001
Lignane secoisolariciresinol (mg)	−0.001	0.000	−0.357	0.005
Cadaverine in feces (*R*^2^ = 0.1, ANOVA *p* = 0.021)				
Constant	10.148	0.403		0.06
Wholegrain servings	0.476	0.200	0.316	0.021

B = unstandardized coefficient; SE B = standard error of B; β = standardized coefficient.

## Data Availability

The original contributions presented in the study are included in the article, further inquiries can be directed to the corresponding author.
